# Accounting for sex differences as a continuous variable in cancer treatments

**DOI:** 10.21203/rs.3.rs-3120372/v1

**Published:** 2023-06-29

**Authors:** Wei Yang, Joshua B Rubin

**Affiliations:** 1 Department of Genetics, Washington University School of Medicine, St. Louis, MO 63110; 2 Department of Pediatrics, Washington University School of Medicine, St. Louis, MO 63110; 3 Department of Neuroscience, Washington University School of Medicine, St. Louis, MO 63110

## Abstract

The significant sex differences that exist in cancer mechanisms, incidence, and survival, have yet to impact clinical practice. We propose that one barrier to translation is that sex differences in cancer phenotypes resemble sex differences in height: highly overlapping, but distinct, male and female population distributions that vary continuously between female- and male- skewed extremes. A consequence of this variance is that sex-specific treatments are rendered unrealistic, and our translational goal should be adaptation of treatment to the variable sex-effect on targetable pathways. To develop a tool that could advance this goal, we applied a Bayesian Nearest Neighbor (BNN) analysis to 8370 cancer transcriptomes from 26 different adult and 4 different pediatric cancer types to establish patient-specific Transcriptomic Indices (TI). TI precisely positions a patient’s whole transcriptome on axes of mechanistic phenotypes like cell cycle signaling and immunity that exhibit skewing in the cancer population relative to sex-identified extremes (poles). Importantly, the TI approach reveals that even when TI values are identical, underlying mechanisms in male and female individuals can differ in identifiable ways. Thus, cancer type, patient sex, and TI value provides a novel and patient- specific mechanistic identifier that can be used for precision cancer treatment planning.

## Introduction

Significant sex differences in cancer incidence and mortality are recognized to be the norm with most cancers exhibiting male to female incidence ratios ranging from 1.26:1 to 4.86:1 [1]. Recent analysis of over 14 million cases from the Cancer Registry, representing 99.9% of the cancer population of the United States confirmed an overall predominance of male cancer cases [2]. An accompanying analysis of survival data from 3.7 million cases in the Surveillance, Epidemiology, and End Results (SEER) database, representing approximately 28% of the cancer population, confirmed that mortality rates are higher for males compared to females [2]. These clinically important sex differences are concordant with described sex differences in cell biology including response to genotoxic stress [3, 4], DNA repair [5, 6], mutational burden and oncogenic mechanisms [7, 8], metabolism [9, 10], and cell cycle regulation [11–13], as well as in systems biology including: immunity [14], metabolism [15, 16], tissue repair [17, 18], and longevity [19, 20]. This suggests that therapies for all cancer patients may be advanced by a realistic translation of sex differences into clinical practice.

The first obstacle is our incomplete understanding of how the genetic, epigenetic, and hormonal foundations of sexual differentiation mechanistically interact with cancer hallmark pathways during tumorigenesis, progression, and response to treatment. A second major obstacle is the nature of biological sex differences. Most sex differences are not dichotomous or sexually dimorphic, like ovaries versus testes. Instead, most sex differences are more akin to height, a complex trait that varies continuously between the shortest females and the tallest male, and is intermediate for most people [21]. Thus, investigating sex effects in cancer is complicated by the varying magnitude of interactions between chromosomal and gonadal sex, and the multiple other cellular, tissue, and body-wide systems that determine an individual’s cancer phenotype. A third obstacle are the recognized ambiguities and inequities in current usage of terms like male, female, men, and women. In this study, we define female and male individuals and cases as XX/ovaries and XY/testes respectively. We recognize that this dichotomy excludes some, e.g., those with sex chromosome aneuploidies, and relies on clinical annotations that may be incorrect at times. In our applications, we think this nomenclature is most accurate and inclusive.

While most individuals possess intermediate heights between the extremes or poles of chromosomal and gonadal sex effects on height determination, the mechanisms underlying an intermediate height may differ in females that taller than the female pole height compared to males who are shorter than the male pole height. Similarly, targeting even similar cancer phenotypes in males and female may benefit from chromosomal and gonadal sex-informed treatment plans.

We sought a method by which to identify mechanisms underlying cancer phenotypes that varied as a function of chromosomal and gonadal sex. To do this, we used a Bayesian Nearest Neighbor (BNN) analysis to identify the poles of male and female transcriptional phenotypes in pediatric and adult cancers. We calculated a patient-specific Transcriptomic Index (TI) value based on the BNN analysis that precisely located individuals between the female (smallest TI values) and male (highest TI values) poles. With this analysis, we determined that most cancer diagnostic groups exhibit transcriptomic variance that correlates to chromosomal/gonadal sex and that cell cycle regulation and immunity/inflammation are the pathways most frequently associated with the male and female poles, respectively. Further, we identified differing mechanisms associated with midrange TI values in female and male patients. We conclude that even when males and females exhibit overlapping phenotypes, the mechanisms underlying that phenotype can differ. This is consistent with published analyses demonstrating that even when genes are equally expressed in males and females, they can exhibit different correlations to cancer mechanisms, treatment responses, and survival [22, 23]. We expect that the TI approach will advance sex differences laboratory and clinical research and provide a paradigm for using an individual’s entire transcriptome for planning their individualized cancer treatment.

## Methods

The use of publicly accessible human datasets for research has been approved by the Washington University Institutional Review Board (IRB# 201102299).

### Inferring Transcriptomic Sex Index (TSI) Using Bayesian Nearest Neighbors

To identify sex-biased gene expression patterns in cancer, we first downloaded the TCGA pan-cancer transcriptome data (gene expression RNAseq - Batch effects normalized mRNA data) from https://pancanatlas.xenahubs.net, the Kids First neuroblastoma data from dbGaP (https://www.ncbi.nlm.nih.gov/projects/gap/cgi-bin/study.cgi?study_id=phs001436.v1.p1) and the Children’s Brain Tumor Network brain tumor data from https://cbtn.org. Sample sizes and number of genes are listed in Table 1. We excluded non-malignancies, cancer types with highly skewed numbers of male or female cases, and those cancers with low incidence (<45 cases in the datasets). The remaining 26 adult and 4 pediatric cancers have sample sizes ranging from 45 to 572, with male samples comprising 27.2% to 84.2% of each cancer type. The total cases examined were 8370 (4927 Males (58.9%), 3443 Females (41.1%)).

Next, we sought to define the Transcriptomic Index (TI) as the Bayesian posterior probability of predicting a patient’s sex from the nearest neighbors based on transcriptomic Euclidean distances. In our previous study we used the Joint and Individual Variance Explained (JIVE) approach to categorically assign transcriptomes to sex-specific subtypes of glioblastoma [23]. An advantage of the Bayesian Nearest Neighbor (BNN) algorithm is that it can infer “breakpoints” between local groupings of nearest neighbors and estimate individual TI values for any transcriptome along a continuous spectrum of values as a Bayesian posterior probability using that transcriptomes’ local neighbors [24].

Following the notations from [24] we denote the target point for predicting patient sex as $x_{\tau }$, and the available training data as $x_{0},x_{1},\cdots ,x_{\tau -1}$, ordered by distance to $x_{\tau }$, with $x_{0}$ being the most distant point from the target. Over a partition ρ of the ordered data points, assume the data is independent and identically from a Bernoulli distribution P(x∣ρ)~Ber(θ), i.e., $P\left(x_{i}=1|i\in \rho \right)=\theta$, with $P\left(x_{i}=0|i\in \rho \right)=1-\theta$, with x=1 indicating the sample being male and x=0 indicating female. The conjugate prior of the Bernoulli distribution is a Beta distribution, θ~Beta(α,β). Moreover, to model the partition ρ, we use $k_{i}$ to denote the number of neighbors in the same partition before sample i when moving from $x_{0}$ towards $x_{\tau }$. Starting from the farthest point, we have

$$p\left(k_{0}=0\right)=1$$

When observing a new datum and moving closer towards the target, we either have a breakpoint and start with a new partition with a certain probability $p_{\gamma }$, or extend the previous partition by 1 at a probability of $1-p_{\gamma }$, i.e.,

$$p\left(k_{i}=0|k_{i-1}\right)=p_{\gamma }$$

$$p\left(k_{i}=k_{i-1}+1|k_{i-1}\right)=1-p_{\gamma }$$

With the above assumptions, we can recursively calculate the joint probability $p\left(k_{i},x_{0},\cdots ,x_{i}\right)$ starting from $x_{0}$ as,

$$p\left(k_{i}=k_{i-1}+1,x_{0},\cdots ,x_{i}\right)=p\left(k_{i-1},x_{0},\cdots ,x_{i-1}\right)p\left(x_{i}|k_{i},x_{0},\cdots ,x_{i-1}\right)p_{\gamma }$$

$$p\left(k_{i}=0,x_{0},\cdots ,x_{i}\right)=p\left(x_{i}|k_{i}=0\right)\sum_{k_{i-1}}p\left(k_{i-1},x_{0},\cdots ,x_{i-1}\right)\left(1-p_{\gamma }\right)$$

In these equations, we can have $p\left(x_{i}|k_{i},x_{0},\ldots ,x_{i-1}\right)$ and $p\left(x_{i}|k_{i}=0\right)$ directly calculated from the Bernoulli distribution. After estimating the joint probabilities, we can easily calculate $p\left(k_{i}|x_{0},\cdots ,x_{i}\right)$ and therefore the final Bayesian posterior probability $p\left(x_{\tau }|x_{0},\cdots ,x_{\tau -1}\right)$ by integrating over the distribution of the number of neighbors $k_{\tau }$. More details of the algorithm for estimating the model could be found from [24]. When applying the above Bayesian model, we used priors α=10 and β=10 for the Beta priors, and $p_{\gamma }=0.05$ for the breakpoint probability.

The TI value, calculated using BNN posterior probability, helps us evaluate the enrichment of female and male samples among nearby neighbors with similar expression profiles. A value close to 1 indicates the vicinity is highly enriched with male samples, while a value close to 0 indicates enrichment with female samples. In our analysis, TI values were calculated separately for each cancer type.

### Downstream analysis

After estimating TI for each patient, the association between TI and gene expression was assessed. Genes with expression positively associated with XY/testes TI values were identified as male - skewed genes, while genes with expression negatively associated with male TI values were identified as female - skewed genes. Linear regression was used for testing gene associations and variable correlations. Enrichment of male and female skewed genes were tested in MSigDB hallmark gene sets using one-sided Fisher’s exact test. Multiple test corrections were performed using the Benjamini-Hochberg FDR Procedure for gene association test and pathway analysis. All measures in the analysis were taken from distinct samples of the involved subjects.

## RESULTS

We previously applied the Joint and Individual Variance Explained (JIVE) algorithm to decompose male and female glioblastoma transcriptome data into components shared among males and females, and those unique to each sex [23]. This allowed us to identify “sex-specific” gene expression patterns and sex-based molecular subtypes of GBM. While informative, the JIVE approach is limited in clinical utility because of the requirement for categorical assignment of gene expression to “male-specific”, “female-specific”, and shared components. “Sex” as a categorical variable has limited utility when investigating sex differences or attempting to stratify individual patients for sex-informed treatments. Thus, we sought to develop a method for generating individual patient-specific values along an axis that traversed between female and male cancer transcriptional “poles”.

**Figure 1:**
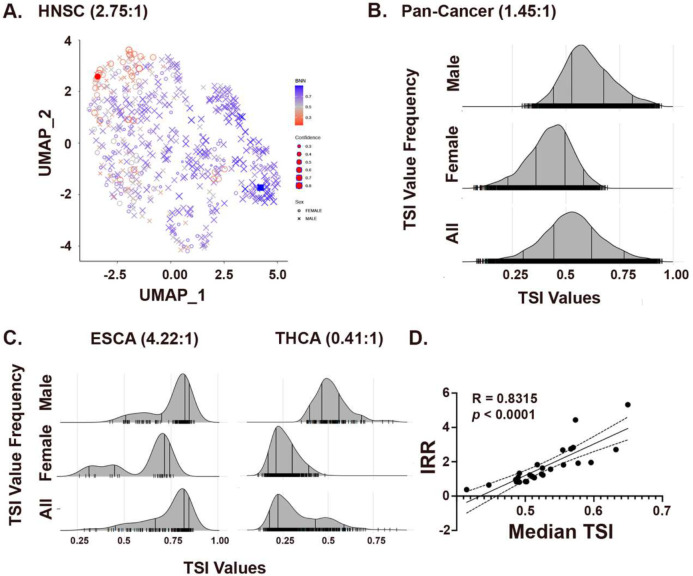
Cancer transcriptomes exhibit skewing by sex. (**A**) UMAP of 566 HNSC transcriptomes clustered by similarity. Male:Female Incidence rate ratio is shown. Male (blue X’s) and female (Red circles) distribute throughout the transcriptional space. Local enrichments for male and female transcriptomes were recognized and quantified to define female (filled red circle) and male (filled blue square) poles of gene expression. BNN value is color-coded and confidence in the posterior probability is indicated by symbol size as indicated. (**B**) Ridge plots of the TI value distributions for Male, Female, and All patients from the PANCAN data (7881 total, 4668 M, 3213 F., M:F IRR = 1.45:1) (**C**) Ridge plots for TI population distributions for Esophageal Carcinoma (ESCA, M/F IRR = 4.22) and Thyroid Carcinoma (THCA, M/F IRR = 0.41) illustrate the correlation between IRRs and median TI values for the 26 adult cancers. (**D**) Regression analysis of IRR vs. Median TI values. Shown is the best fit and 95% confidence intervals. R and *p* values are shown.

We created UMAPs from the transcriptomes of each cancer based on similarities in gene expression. The head and neck squamous carcinoma (HNSC) UMAP, illustrates the process of identifying female and male poles in the data (Fig 1A). We discovered local areas in the UMAP where samples were primarily male (blue exes) or female (red circles). We quantified the local chromosomal/gonadal sex enrichment and used them as poles (filled red circle and filled blue square) to detect skewing in cancer transcriptomes. We defined TI as the Bayesian posterior probability of predicting the sex of a patient based on their individual transcriptomic similarity to the female and male poles. Thus, this index value reflects local transcriptomic patterns. A Bayesian algorithm [25] was used to infer “breakpoints” between local and remote samples. High TSI values are locally enriched in male cases, while low TSI values are locally enriched in female cases.

We derived TI values for all individual cases and median TI values in every cancer population separately (**Supplemental Figure 1)**. Then, we combined all 7881 adult TI values to create a pan-cancer TI population distribution (Fig 1B). As can be seen, the female and male values are skewed. Further, cases with TI values below 0.25 are exclusively female while only males have values above 0.75. These cases represent the female and male population poles, respectively. It is also clear from the data that a large fraction of the cancer population possesses TI values between 0.25 and 0.75. We can expect that as female cases approach TI values of 0.5, they represent a changing balance between pole effects that will mechanistically differ from those in male cases approaching the same TI value.

As expected, median TI values for each cancer type positively correlated with their incidence rate ratios (IRR, M:F) as calculated from these datasets. As illustrated in Fig 1C, esophageal carcinoma (IRR = 4.22) and thyroid carcinoma (IRR = 0.41) exhibit median TI values of (0.65) and (0.41), respectively. Similarly, the other cancers with IRRs of less than 1 (sarcoma, adrenocortical carcinoma, diffuse large B cell lymphoma, thyroid carcinoma) exhibit median TI values of less than 0.50 (**Supplemental Table 1**). Regression analysis of IRR versus median TI identified a significant correlation between the two (Fig 1D). Thus, TI value distributions are concordant with sex differences in individual cancer IRRs. Importantly, TI value indicates that many male individuals with IRR < 0.5 cancers exhibit individual TI values that are shifted towards the female pole and that many female individuals with IRR > 0.5 cancers exhibit individual TI values that are shifted towards the male pole. This does not mean that some females are “male-like”, and some males are “female-like” and highlights can kind of ambiguity that can arise from the use of these terms. It simply describes a phenotype like a tall female or a short male.

Together, these data suggest that chromosomal and gonadal sex effects on gene expression are pervasive and interact with cancer type-specific effects on gene expression to produce individual transcriptomes. Thus, we next sought to identify the genes and pathways that define the high and low TI poles. We did so by looking for consistency in sex-skewed mechanisms across cancer types using the 7881 adult and in parallel, the 1069 pediatric cases. Those genes with the greatest effect on low and high TI values were identified by performing association analysis between TI and gene expression. Genes that were significantly (FDR<0.05) associated with high TI were identified as “male-skewed genes”, while those negatively associated with high TI were identified as “female - skewed” (**Supplemental Table 2**).

**Figure 2:**
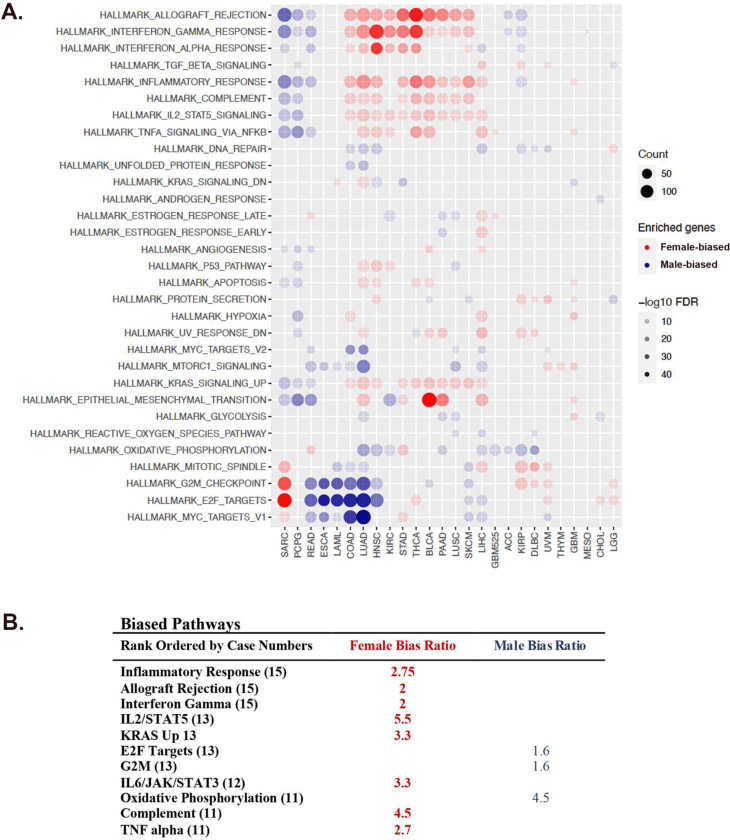
Most Cancers exhibit sex - skewed hallmark pathway activation. (**A**) Genes with the greatest effect on low (female pole) and high (male pole) TI values were identified. Cancer Hallmark Pathway analysis of pole-associated genes revealed a predominant polarization pattern involving cell cycle regulation and oxidative phosphorylation at the male pole (blue circles) and multiple inflammatory/immunity pathways at the female pole (red circles). Gene counts (count) are symbolized by the size of the circles and False Discovery Rates (FDR) by the saturation of the fill as indicated in the legends. (**B**) Frequency of pathway skewing is listed in rank order of cases involved (in parentheses) and the ratio of involved female (red text) to male cases (blue text).

Cancer Hallmark Pathway analysis across cancer types indicated that most hallmark pathways [26] exhibit skewing in gene expression and revealed several patterns of transcriptome polarization (Figure 2A and B). Seventeen of the 26 cancer types were enriched for genes involved in oxidative phosphorylation and/or cell cycle regulation at the male pole. Twelve of the 26 cancers were enriched for genes involved in inflammation and immunity at the female pole (Figure 2A and B). Mesothelioma was the only cancer without evidence of polarization. Interestingly, sarcoma differed from the predominant polarization patterns such that male cases were enriched for inflammation/immunity signatures and female cases for cell cycle regulation. This emphasizes the need to interpret TI values within the context of cancer type and patient sex.

These data indicate that varying degrees of chromosomal/gonadal sex - correlated gene expression exist across cancer types and that a predominant shared pattern between multiple cancers involves skewed gene expression in cell cycle regulation versus inflammation/immunity pathways, which are known to be strongly sex-biased in action [11–14]. Thus, we conclude that TI value can successfully localize individual cancer cases along axes that traverse between chromosomal/gonadal sex poles in targetable mechanisms like cell cycle regulation and immunity/inflammation.

As much of sexual differentiation is completed *in utero*, we hypothesized that similar skewing in TI would be evident in pediatric cancers. We applied the same strategy to the analysis of two pediatric transcriptome datasets: the Gabriella Miller Kids First Pediatric Research Program (Kids First (KF)), which included 209 neuroblastoma patients and the Children’s Brain Tumor Network (CBTN), which included 865 patients comprised of 101 high grade glioma, 105 medulloblastoma, 79 ependymoma, and 214 low grade glioma cases (Table 1). From the KF data, 6330 male - skewed genes and 6089 female - skewed genes were identified (FDR<0.05, **Supplemental Table 3**). From the CBTN data, 3063 male - skewed genes and 2062 female - skewed genes were identified (FDR<0.05, **Supplemental Table 4**). There were 1126 shared male - skewed genes, and 742 shared female - skewed genes between the two datasets (both with p<2.2e-16, **Supplemental Table 5**).

**Figure 3:**
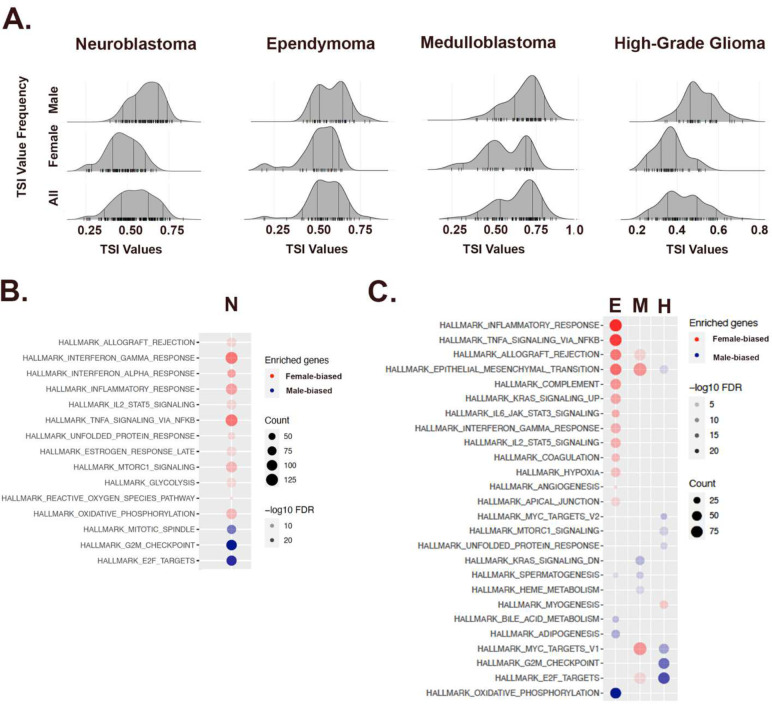
Pediatric neural tumors also exhibit sex - skewed gene expression. (**A**) Ridge plots for neuroblastoma and the three most common malignant brain tumors of childhood (489 total, 259 M, 230 F., M:F IRR = 1.13:1) demonstrating sex -skewed TI population distributions. (**B**) Cancer Hallmark Pathway analysis of those genes that exerted the greatest effects on the male and female poles for each cancer. A predominant polarization pattern is identified with inflammatory/immunity pathways associated with the female pole (red circles) and cell cycle regulatory pathways associated with the male pole (blue circles). Gene counts (count) are symbolized by the size of the circles and False Discovery Rates (FDR) by the saturation of the fill as indicated in the legends.

Like the adult cancers, these pediatric cancers exhibited biased distributions of TI value (Figure 3A, **Supplemental Figure 2**). Neuroblastoma (IRR = 1.11) is strongly polarized and again, those cases with high TI values were enriched for cell cycle regulation while those associated with low TI values were enriched for inflammation and immunity (Figure 3B). The CBTN brain tumor data includes the diverse cancer types common in pediatric neuro-oncology. We focused our analysis on the most common and malignant pediatric brain tumors. Ependymoma (IRR = 1.5) exhibited the strongest polarization, in which low TI values were enriched for inflammation and immunity and oxidative phosphorylation was strongly correlated with high TI values (Figure 3C). The most common malignant brain tumor of childhood is medulloblastoma (IRR: 1.8:1) [27]. The strongest association in medulloblastoma was between low TI value and cell cycle regulation, reminiscent of what was observed for adult sarcomas. In pediatric high-grade glioma (IRR ≈ 1), high TI values were strongly correlated with cell cycle regulation, while there were no distinct gene expression patterns associated with low TI value cases. Thus, like adult cancers, pediatric cancers exhibit chromosomal/gonadal sex skewed gene expression that varies in magnitude and involved pathways, indicating that varying interactions occur between chromosomal/gonadal sex and tumor type in both adult and pediatric cancers. Importantly, skewed gene expression and pathway activation are evident even when the incidence ratios of cancer types (e.g., pediatric high-grade glioma) near equivalence. Therefore, individuals with any cancer type can be more extensively phenotyped for personalized approaches to treatment using a TI analysis than without.

**Figure 4:**
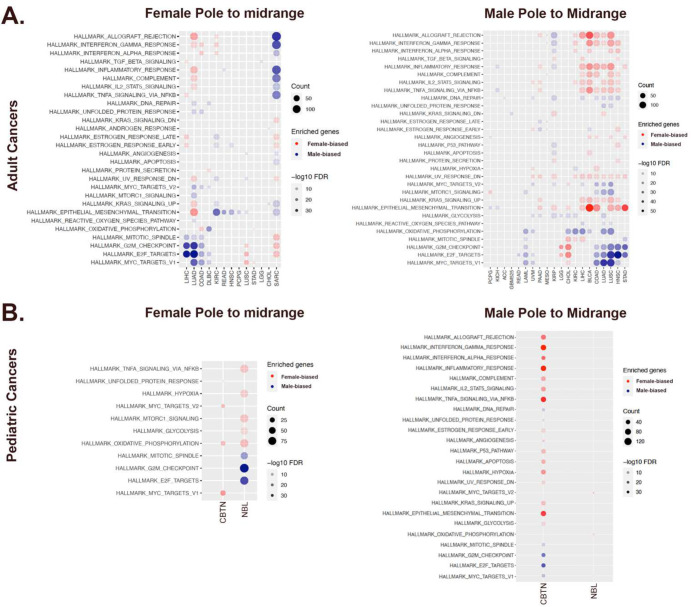
Mid-range TI values exhibit distinct pathway signatures relative to the sex – defined poles. (**A**) (Left Panel) Heatmap of pathway activation signatures underlying changes in midrange TI values for female adult cancers relative to their pole. (Right Panel) Heatmap of pathway activation signatures underlying changes in midrange TI values for male adult cancers relative their pole. (**B**) (Left Panel) Heatmap of pathway activation signatures underlying changes in midrange TI values for pediatric female cancers relative to their pole. (Right Panel) Heatmap of pathway activation signatures underlying changes in midrange TI values for pediatric male cancers relative their pole. For all panels, changes in male (blue circles) and female (red circles) signatures are indicated. Gene counts (count) are symbolized by the size of the circles and False Discovery Rates (FDR) by the saturation of the fill as indicated in the legends. Only cancer types and pathways with significant change are shown.

Cancer patients with extremes of high and low TI value might be approachable with something akin to sex-specific treatments as they approach dichotomy in phenotype. However, TI value for most patients lies between the poles. Therefore, we expected that their transcriptomes would exhibit both female and male skewed components. We hypothesized that for female cases, translation along the TI axis from < 0.25 to midrange values would involve decreased female - skewed effects and/or increased male - skewed effects. We predicted that the opposite would be the case for male cases with midrange TI value compared to those with TI > 0.75. If this proved to be the case, we expected this could serve as a tool for stratification for chromosomal/gonadal sex – informed treatments, even for those of differing sex with identical TI value. To address this hypothesis, we first compared the PANCAN transcriptomes of all cases with midrange TI value to those with transcriptomes closer to their respective poles. We then performed pathway analysis to determine which pathways were altered relative to their poles. Several clear patterns of change in different cancer types emerged. Across cancer types, most female cases, exhibited a loss of the inflammatory/immunity signatures (Figure 4A). Female cases of four cancer types (LIHC, LUAD, COAD, DLBC) exhibited a gain in cell cycle regulatory signature. Male cases of seven cancer types (PRAAD, KIRC, LIHC, BLCA, COAD, LUAD, LUSC), exhibited a clear increase in the inflammation/immunity signature (Figure 4A). There were mixed patterns of gains and losses of the other “pole-defining” pathways, such as cell cycle regulation and oxidative phosphorylation, across cancer types. In contrast, female sarcoma cases with midrange TI value cases exhibited gains in female - skewed cell cycle regulatory and male - skewed inflammation/immunity patterns of gene expression. Midrange male sarcoma cases exhibited no significant change in these pathways. Finally, several cancer type-specific changes in intracellular epithelial-to-mesenchymal transition (EMT) and key intracellular signaling pathways such as MYC, MTORC1, or KRAS, occurred in female and male cases with midrange TI values. Together, these data emphasize the potential of this approach for identifying chromosomal/gonadal sex-skewed actions in targetable pathways, even for those with overlapping mid-range TI value.

Next, we performed the same analysis in the pediatric datasets. Like the PANCAN analysis, translation away from their respective poles to midrange TSI values occurred concomitantly with a shift in pole - defining pathway involvement (Figure 4B). For increased power, we combined all malignant brain tumor cases for this analysis. In the CBTN brain tumor data, we found that female cases with midrange TI value exhibited decreased MYC targets (V1 and V2) and oxidative phosphorylation. Male CBTN cases with midrange TI value, exhibited significant changes in almost all hallmark pathways with gains in both the female - skewed inflammatory signature and loss of the male - skewed cell cycle and oxidative phosphorylation signatures.

In the KF Neuroblastoma data, female cases with midrange TI value exhibited a strong acquisition of a male - skewed cell cycle regulatory signature as well as decreased female - skewed inflammation/immunity and metabolism signatures (Figure 4B). No skewed pathway signature changes were detectable in the male midrange TI value cases. Together, these results support the hypothesis that midrange TI value can be associated with different molecular pathway activation profiles in female and male individuals with different cancer diagnoses. Thus, even when similar in transcriptomic phenotype, particular subsets of female and male cancer patients may benefit from chromosomal/gonadal - sex informed therapies.

## Discussion

Sex effects in cancer incidence, treatment, and survival are commonplace [2]. Like any other significant difference in cancer phenotypes, understanding the mechanistic basis for sex differences holds promise for improving outcomes for all. Sex-adaptations to treatment are complicated by the nature of sex differences. Most sex differences are not as dichotomous as a peacock’s tail. Instead, individuals are a unique balance of sex chromosome and hormone actions. This suggests that the optimal translation of sex differences in cancer biology will require recognizing sex effects as continuous variables and making measurements of the individual balance between sex chromosome and hormone – skewed actions in each patient.

Here, we showed that relative male- and female- biased actions can be quantified through a Bayesian Nearest Neighbor-based derivation of a Transcriptomic Index. The value of this approach is it uses the entire patient-specific tumor transcriptome, as well as a realistic treatment of sex differences as continuous variables, to make predictions regarding the relative contributions that targetable sex chromosome and hormone - skewed actions make to each individual patient’s cancer biology.

**Figure 5:**
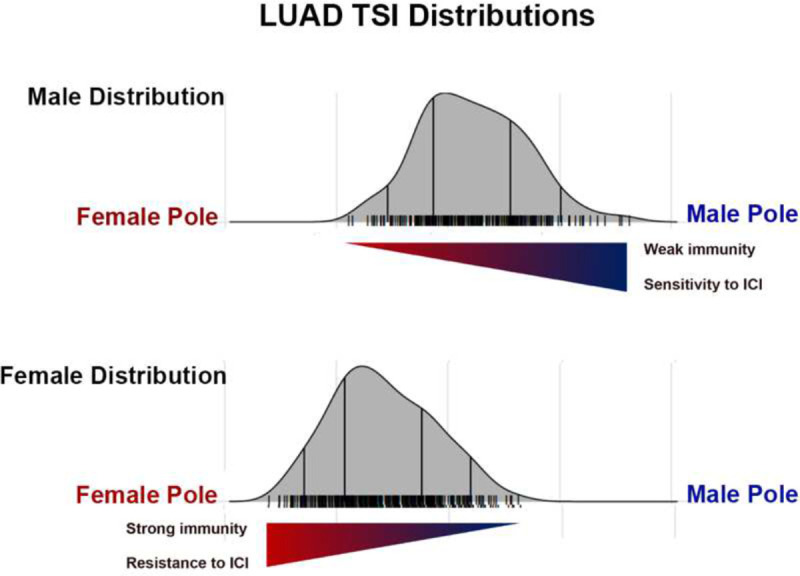
Example application of TI in patient treatment stratification. Pictured are the TI distributions for female (top panel) and male (botto panel) lung adenocarcinoma (LUAD) patients. Females exhibited a strong immunity/inflammation signature and in clinical trials, are resistant to immune checkpoint inhibition (ICI). In contrast, male LUAD patients do not exhibit an inflammation/immunity signature and are responsive to ICI. If male and female patients were stratified for immune checkpoint inhibition treatment, the most likely males to respond to treatment would be those with the highest TI, those nearest the male pole. Female patients most likely to respond to treatment would also be those with the highest TSI values, those furthest from the female pole.

To do this, we first identified female and male extremes in cancer transcriptional phenotypes and then, the genes and pathways underlying these poles. We observed several different polarization patterns, highlighting that chromosomal and gonadal sex interacts in variable ways with differing cancer mechanisms, i.e., cells of origin, specific oncogenic events, as well as their tissue and systems biology. The most common polarizations occur around cell cycle regulation and inflammation/immunity in both adult and pediatric cancers. This serves as validation for this approach as both mechanisms are already known to exhibit substantial sex differences under normal conditions [11–14]. As both pathways are targetable with available therapeutics [28, 29], it is interesting to consider how the TI approach might inform stratification for treatment. As an illustration, clinical experience indicates that females exhibit a smaller survival benefit from immune checkpoint inhibition (ICI) than males [30–32]. Thus, it would be reasonable to hypothesize that the immune signature associated with the lowest TI values is one of resistance to ICI. If so, then females with low TI values would be predicted to be less responsive to ICI than those with higher TI values.Concordantly, males have been shown to be more responsive to ICI and therefore, high TI values may be a biomarker for ICI sensitivity and males with lower TI values may be more resistant to ICI than those with higher TI values (Figure 5). In this way, sex, cancer type, and TI values might more precisely stratify patients for ICI, or by analogy other targetable pathways.

Thus, we can no longer focus on questioning whether chromosomal/gonadal sex differences matter in cancer. Sex differences in incidence, response to standard treatments, and survival, all strongly argue that they do. The question now is, how do we use the continuously varying nature of sex differences for treatment planning, patient stratification, and analysis of laboratory and clinical research results. As the TI analysis enriches patient-specific phenotyping, we expect that its use can enhance personalized approaches to treatment by realistically accounting for sex effects in cancer. It is important to note that substantial sex differences exist in normal aging [33] and multiple conditions such as cardiovascular, rheumatological, psychiatric, and other diseases with substantial impact on the human condition [34]. The TI approach is readily adaptable to these conditions as well.

Finally, accounting for biological differences that arise through sexual differentiation and the profound effects of all the downstream consequences of having two X-chromosomes versus one X and one Y chromosome, is complicated by the ambiguities and inequities that arise with terms like male, female, men, women, alone or when combined with terms like -specific, -differences, -biased, -effect, etc. While we have used these terms, we now propose that low and high TI values can be utilized in a manner like height. While height is significantly skewed in female versus male populations, the term itself carries no sex or gender implications. Similarly, other phenotypes such as carbohydrate- versus fatty acid- centered metabolism exhibit population skewing in female versus male individuals, but we need not refer to this as female - versus male - metabolism. Instead, we can recognize that targeting metabolism may require understanding the ways in which a carbohydrate-based cellular metabolism can differ in females versus males.

## Figures and Tables

**Table 1:** Case Data

PANCAN Types	genes	Male	Female	Total	% Male

Thyroid Carcinoma (THCA)	20531	157	415	572	27.4
Adrenal Cortical Carcinoma (ACC)	20531	31	48	79	39.2
Pheochromocytoma and Paraganglioma (PCPG)	20531	84	103	187	44.9
Sarcoma (SARC)	20531	120	145	265	45.3
Diffuse Large B-Cell Lymphoma (DLBC)	20531	22	26	48	45.8
Lung Adenocarcinoma (LUAD)	20531	265	311	576	46.0
Cholangiocarcinoma (CHOL)	20531	22	23	45	48.9
Thymoma (THYM)	20531	63	59	122	51.6
Colon Adenocarcinoma (COAD)	17507	257	235	492	52.2
Rectal Adenocarcinoma (READ)	17507	90	80	170	52.9
Acute Myeloid Leukemia (LAML)	16765	93	80	173	53.8
Low Grade Glioma (LGG)	20531	291	238	529	55.0
Pancreatic Adenocarcinoma (PAAD)	20531	101	82	183	55.2
Uveal Melanoma (UVM)	20531	45	35	80	56.3
Kidney Chromophobe (KICH)	20531	52	39	91	57.1
Glioblastoma 525 (GBM525)	8720	320	205	525	61.0
Skin Cutaneous Melanoma (SKCM)	20531	293	180	473	61.9
Glioblastoma (GBM)	20531	107	59	166	64.5
Stomach adenocarcinoma (STAD)	16765	291	159	450	64.7
Kidney Renal Clear Cell Carcinoma (KIRC)	20531	398	208	606	65.7
Liver Hepatocellular Carcinoma (LIHC)	20531	280	143	423	66.2
Bladder Carcinoma (BLCA)	20531	311	116	427	72.8
Kidney Renal Papillary Cell Carcinoma (KIRP)	20531	236	87	323	73.1
Head and Neck Squamous Cell Carcinoma (HNSC)	20531	415	151	566	73.3
Lung squamous cell carcinoma (LUSC)	20531	408	144	552	73.9
Mesothelioma (MESO)	20531	71	16	87	81.6
Esophageal Carcinoma (ESCA)	19076	165	31	196	84.2

Total (PANCAN)		4668	3213	7881	59.2

**Pediatric Types**	**genes**	**Male**	**Female**	**Total**	**% Male**

Neuroblastoma	22586	104	105	209	49.8
ATRT	25076	12	12	24	50.0
Craniopharyngioma	25076	19	15	34	55.9
DNET	25076	13	9	22	59.1
Ependymoma	25076	44	32	76	57.9
Ganglioglioma	25076	24	17	41	58.5
High grade glioma	25076	43	56	99	43.4
Low grade glioma	25076	116	98	214	54.2
Medulloblastoma	25076	68	37	105	64.8
PNET	25076	6	11	17	35.3
Others	25076	128	100	228	56.1

Total Pediatric		577	493	1069	54.0
